# Weldability of Underwater Wet-Welded HSLA Steel: Effects of Electrode Hydrophobic Coatings

**DOI:** 10.3390/ma14061364

**Published:** 2021-03-11

**Authors:** Jacek Tomków

**Affiliations:** Division of Welding Engineering, Faculty of Mechanical Engineering and Ship Technology, Gdańsk University of Technology, Gabriela Narutowicza 11/12, 80-233 Gdańsk, Poland; jacek.tomkow@pg.edu.pl

**Keywords:** high-strength low-alloy steel, underwater welding, wet welding, weldability, hydrophobic coating

## Abstract

The paper presents the effects of waterproof coatings use to cover electrodes on the weldability of high-strength, low-alloy (HSLA) steel in water. With the aim of improving the weldability of S460N HSLA steel in water, modifications of welding filler material were chosen. The surfaces of electrodes were covered by different hydrophobic substances. The aim of the controlled thermal severity (CTS) test was to check the influence of these substances on the HSLA steel weldability in the wet welding conditions. The visual test, metallographic tests, and hardness Vickers HV10 measurements were performed during investigations. The results proved that hydrophobic coatings can reduce the hardness of welded joints in the heat-affected zone by 40–50 HV10. Additionally, the number of cold cracks can be significantly reduced by application of waterproof coatings on the filler material. The obtained results showed that electrode hydrophobic coatings can be used to improve the weldability of HSLA steel in underwater conditions.

## 1. Introduction

Each year the usage of high-strength, low-alloy (HSLA) steels increases. These materials are characterized by their mechanical properties, which allow the weight of structures to be reduced [1]. They are used as the elements of many welded structures, e.g., bridges, wind farms, and building skeletons [2,3]. Additionally, HSLA steels are the most often used materials for marine and offshore structures, which are in direct contact with water, e.g., ships, wharfs, pipelines, and tanks [4]. These structures may undergo damage that has to be repaired in underwater conditions [5,6].

There are three different methods of underwater welding. The first is dry welding, in which the welder and the process area are isolated from the surrounding water by special chambers [7,8]. The second method is local cavity welding, whereby the welding area and welding arc are located in a small chamber without water, however the welder–diver is located in the aqueous environment [9]. The last method is wet welding [10], whereby the welder and the joining area are in direct contact with the water [11]. Due to the low cost of this process and it requiring the cheapest equipment, wet welding is the most widely used underwater welding processes. The most often used welding processes in wet welding are flux-cored arc welding (FCAW) [12] and manual metal arc (MMA) welding [13]. Despite its wide range of applications, there are many problems during wet welding. From a technological point of view, the most important is the instability of the welding arc. This lowers the quality of the welded joints. Wang et al. [14] stated that stability of the welding arc depends on the welding parameters. They found that lower welding current values increased the stability of the welding arc in wet welding conditions. However, the quality of welded joints was greater with higher current values. Moreover, it was stated that the welding arc stability was much lower than during welding in air. The instability of the welding arc in underwater conditions resulted from gas bubbles being created in the water, which was proven by Xu et al. [15]. Such bubbles are filled with hydrogen, which can also penetrate into welded joints. The diffusible hydrogen content in the deposited metal lowers the quality of underwater wet-welded joints [16]. Tomków et al. [17] stated that the diffusible hydrogen content is 50% higher in underwater conditions than during air welding. The hydrogen content does not change significantly when varying the water depth, which was proven by Klett et al. [18]. The next problem that occurs in wet welding conditions is the formation of brittle structures in the heat-affected zone (HAZ) [19]. Wang et al. [20] proved that the brittle microstructure in the HAZ of their specimen resulted from the high cooling rate during underwater welding. In the same investigation, the authors stated that such brittle structures result in deteriorated corrosion resistance in the welded joints. Increasing the depth of welding decreases the corrosion rate, as shown by Surojo et al. [21]. 

Existing problems affect the high susceptibility to cold cracking of HSLA steels in water [5,9,10], in turn decreasing the quality of underwater-welded joints. Due to this, the investigations of underwater welding are focused on the possibility of improving the weldability of different grades of steels. One of such method, which is widely used in open-air conditions, is heat treatment [22]. However, aqueous environments do not allow traditional processes to be performed. One method that allows similar results to be obtained is temper bead welding (TBW), which showed positive effects in open-air conditions [23,24]. This method involves depositing one or more welding beads (tempering) on a previously laid bead (tempered), reducing the hardness in the HAZ, which results from microstructural changes caused by the influence of heat from tempering beads. The TBW method was used during wet welding of S460N HSLA steel by Tomków et al. [25]. Investigations were performed with different pitch values (percentage of overlap between two beads). The TBW method applied with the recommended pitch reduced the hardness in the HAZ by 40–50 HV10 in S460N steel. Additionally, the grain size was decreased and the brittle structures were tempered in the HAZ. However, there was a big problem with controlling the required value of the pitch due to the limited visibility and operability during MMA wet welding. Another investigated method that improved the weldability in underwater conditions was ultrasonic-assisted welding. This method is based on creating ultrasonic vibrations in the filler material. Wang et al. [26] proved that this method can improve the stability of the welding arc, improving the quality of FCAW joints. Ultrasonic-assisted welding can improve the mechanical properties of FCAW joints, as found by Chen et al. [27]. However, the high ultrasonic power involved generates cavitation bubbles. The cavitation bubbles produced by ultrasonic-assisted welding are then left in the weld metal and become welding pores. Another disadvantage of the proposed method is the need to use special equipment, which limits the possibility of applying this method in non-laboratory welding conditions. The literature analysis showed that improved methods are still required in order to improved the weldability of HSLA steel.

In welding engineering, protective coatings are often used to improve the properties of welded metals. Coatings are deposited on the surfaces using different welding techniques, such as pad welding, thermal spraying, or plasma deposition [28,29]. Most of the layers improve the mechanical properties of the surfaces [30]. However, the implementation of hydrophobic substances is also a well-known process. Gnedenkov et al. [31] used plasma electrolytic oxidation to improve the welded joint corrosion resistance. The possibility of improving the corrosion resistance by using hydrophobic coatings deposited on metals was also proven by Zhang et al. [32]. The properties of welded joints result from the properties of the filler materials [33,34,35,36]. Following this reasoning, hydrophobic substances are used for modification of welding wires and electrodes. Amaral et al. [37] used polytetrafluoroethylene as a flux ingredient in underwater flux-cored arc pad welding. The experiment showed that the hydrophobic substance, as a part of the flux, decreases the diffusible hydrogen content in the deposited metal. Menezes et al. [38] used polymer-agglomerated electrodes for MMA pad welding in wet welding conditions. The performed experiments showed decreased hydrogen content in the deposited metal and improved quality of the pad welds. The influence of waterproof coatings on the quality of pad welds was investigated by Tomków et al. [39]. It was proven that a waterproof coating affects the quality of layers welded in wet welding conditions. It was stated that the use of paraffin wax decreased the hardness and number of cold cracks in a tap water environment.

The aim of this paper was to study the influence of electrode hydrophobic coatings on the weldability of HSLA steel in water. To the author’s best knowledge, the usage of waterproof coatings during wet welding of joints with fillet welds had not previously been investigated. Different commonly used substances were chosen as hydrophobic coatings. These substances were applied on commercial covered electrodes.

## 2. Materials and Methods

### 2.1. Materials

As a base metal (BM), S460N HSLA steel (12 mm thick) was chosen. This material is characterized by cold cracking in aqueous environments, which was proven in previous investigations [40]. For welding, two grades of covered electrodes (ISO 2560-A: E 38 0 R11); (4.0 mm in diameter) and general-purpose mild steel electrodes (4.0 mm diameter; nearest equivalent E42 2 1Ni RR 51, named “underwater electrodes”) were chosen for underwater welding. Both are made of rutile, which is widely used for wet welding, allowing welds characterized by high ductility [41]. It helps to decrease the susceptibility of the metal to cold cracking. The first electrodes were modified by depositing different hydrophobic substances. The chemical compositions of materials are presented in Table 1. Their mechanical properties are shown in Table 2.

### 2.2. Procedure and Methodology

Investigations were performed in tap water (0.5 m depth) at 20 °C, using the MMA method. Before welding, E38 0 R11 electrodes were modified by surface application on the commonly used hydrophobic substances. In previous investigations carried out on pad welds, different waterproof coatings were tested [39]. Following these investigations, for this research the concrete impregnate, liquid foil, and paraffin wax were chosen. These substances offered the best results in improving the quality of pad welds in wet welding conditions [39]. The surfaces of covered electrodes were coated with hydrophobic substances using the brush painting process. A description of the used hydrophobic substances is presented in Table 3.

**Figure 1:**
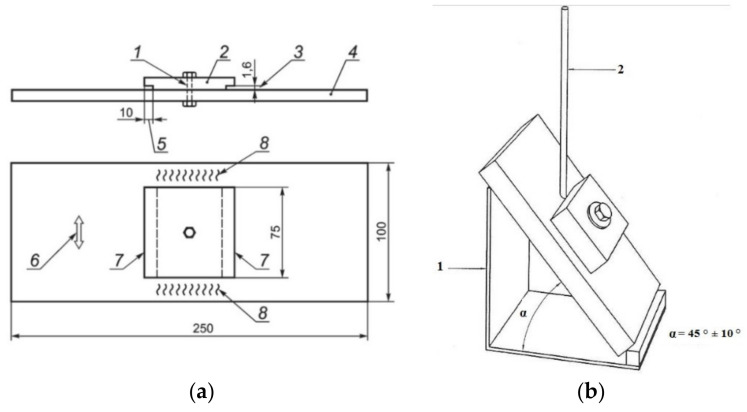
Control thermal severity (CTS) specimen: (**a**) scheme of CTS test; 1 –hole 13 mm diameter, 2—top plate, 3—root notch gap, 4—bottom plate, 5—root notch depth, 6 –rolling direction, 7—test welds, 8—anchor welds; (**b**) the location of the specimen during welding; 1—jig, 2—covered electrode.

For this research, the control thermal severity (CTS) test was selected, which resulted from the fact that fillet welds are the most commonly used in underwater-welded structures. Additionally, the CTS test assesses susceptibility to cold cracking in all regions of welded joints. A scheme of the CTS specimens is presented in Figure 1a. The specimen was housed in a special jig. The test welds were deposited symmetrically in the flat position (PA) in a single pass and in a single direction, across the full width of the block (Figure 1b).

The welding process was carried out following the methodology required by the EN-ISO 17642-2:2005 standard [42]. The second test weld was performed 48 h after the first test weld. In total, five CTS specimens were welded—one with the usage of E38 0 R11, one with an underwater electrode, and three with modified covered electrodes (48 h after application of hydrophobic substances). All specimens were welded manually with negative polarity (DC-). The welding speed (Vsp), welding current (I), arc voltage (U), and heat input (ql) were chosen in accordance with the range required by filler material manufacturers. The welding parameters led to changes in the properties of the welded joints [43]. The control of welding parameters is common in air welding conditions [44]. However, in water conditions, changes in heat input occurred, resulting from problems generated by the environment. In the presented investigation, the welding parameters were kept at similar levels in order to obtain the most comparable conditions. The welding parameters are presented in Table 4.

Following the appropriate standard [42], each test weld was examined using a non-destructive test (NDT), namely the visual test (VT), following the EN ISO 17637:2017 standard [45]. The first tests were started 48 h after the second test weld [46]. In the next step, the destructive tests (DTs) were performed. From each test weld, two samples were cut—samples numbered as 1 and 2 from weld no. 1, and samples numbered as 3 and 4 from weld no. 2. Specimens were ground, polished, and etched (Nital 4%). Then, the metallographic macroscopic tests were undertaken following the EN ISO 17639:2013 standard [47] using a Canon EOS 1200D camera (Canon, Tokyo, Japan). This test was started 48 h after NDTs. In the next step, the Vickers HV10 hardness was measured based on the requirements listed in the EN ISO 9015-1:2011 standard [48]. The Sinowon V-10 instrument (Sinowon, Dongguan, China) was used for measurements. At the end of the process, the microscopic test following the requirements listed in the relevant standard [47] was undertaken using an Olympus BX51 light microscope (Olympus, Tokyo, Japan). All tests were performed at 20 °C.

## 3. Results and Discussion

### 3.1. Process Observations

The first differences between electrodes surfaced when different hydrophobic substances were observed during welding. The joining with non-modified E38 0 R11 electrodes (specimen 1) resulted in stability of the welding arc. There were no problems with slag removal. Similar results were observed during welding of specimen 2, which was joined by an underwater electrode. Using the concrete impregnate (specimen 3) led to problems with the welding arc initiation. However, during the process, the arc was stable. No problems were observed during slag removal. The biggest problems occurred during welding of specimen 4 using electrodes coated with liquid foil. There were problems with arc initiation, which burned in an unstable manner during the process. The highest number of gas bubbles was observed during welding of this specimen. This proved the connection between a high number of gas bubbles and instability of the welding arc, which had been previously stated in the literature [15]. Additionally, the water became very contaminated, which affected the visibility of the welder. Welding using electrodes coated with paraffin wax led to instability of the welding arc. However, there were no problems during arc initiation. Moreover, the slag was removed easily. Similar phenomena were observed in previous investigations, which were performed for pad welds [39].

### 3.2. Visual Testing

**Figure 2:**
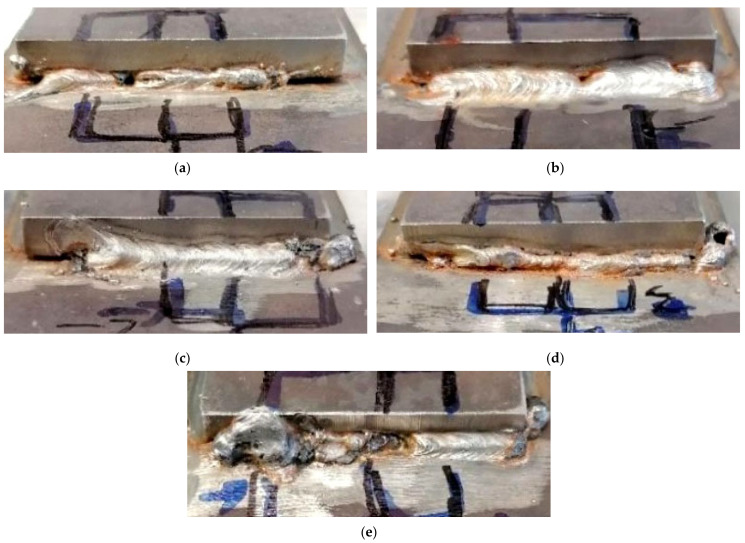
The exemplary results of the visual test (VT): (**a**) weld 1—specimen 1 (non-modified electrode), pores, weld length 60 mm; (**b**) weld 2—specimen 2 (underwater electrode), weld length 70 mm; (**c**) weld 1—specimen 3 (concrete impregnate), spatter, weld length 60 mm; (**d**) weld 2—specimen 4 (liquid foil), lack of fusion, undercuts, shape defects, weld length 70 mm; (**e**) weld 1—specimen 5 (paraffin wax), shape defects, pores, weld length 70 mm.

Exemplary photos of the VT are presented in Figure 2. The lines mark places identified for sample cutting in further investigations. The VT focused on the selection of cutting locations for further investigations, due to the fact that underwater wet welding in most cases is treated as a repair method. Our observations showed differences between specimens performed using electrodes with different surface coatings. Both test welds in specimens 1 and 2 are characterized by their proper geometry. However, pores were observed in one weld in each specimen (Figure 2a,b). Welding of specimen 3 generated excessive spatter (Figure 2c), which was not observed for other specimens. The worst results were observed for the specimen welded using electrodes coated with liquid foil. The width of the weld was two-fold smaller than in other specimens. Additionally, a lack of fusion and undercuts was observed (Figure 2d). The biggest difference between two welds in the same specimen was observed for specimen 5. Weld 2 performed with higher heat input and was characterized by its proper geometry. Moreover, no imperfections were observed. The VT for weld 1 showed many imperfections, such as shape defects and pores, which started in the middle of the weld (Figure 2e). Guo et al. [49] proved that the heat input value is crucial to the quality of offshore welded steel joints. Additionally, in the author’s previous investigations [25], it has been shown that higher heat input values lead to improvement of the quality of S460N steel wet-welded joints. 

### 3.3. Metallographic Macroscopic Testing

**Figure 3:**
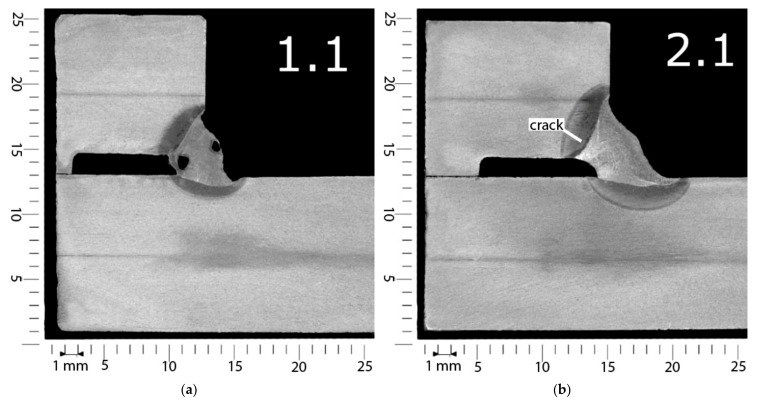

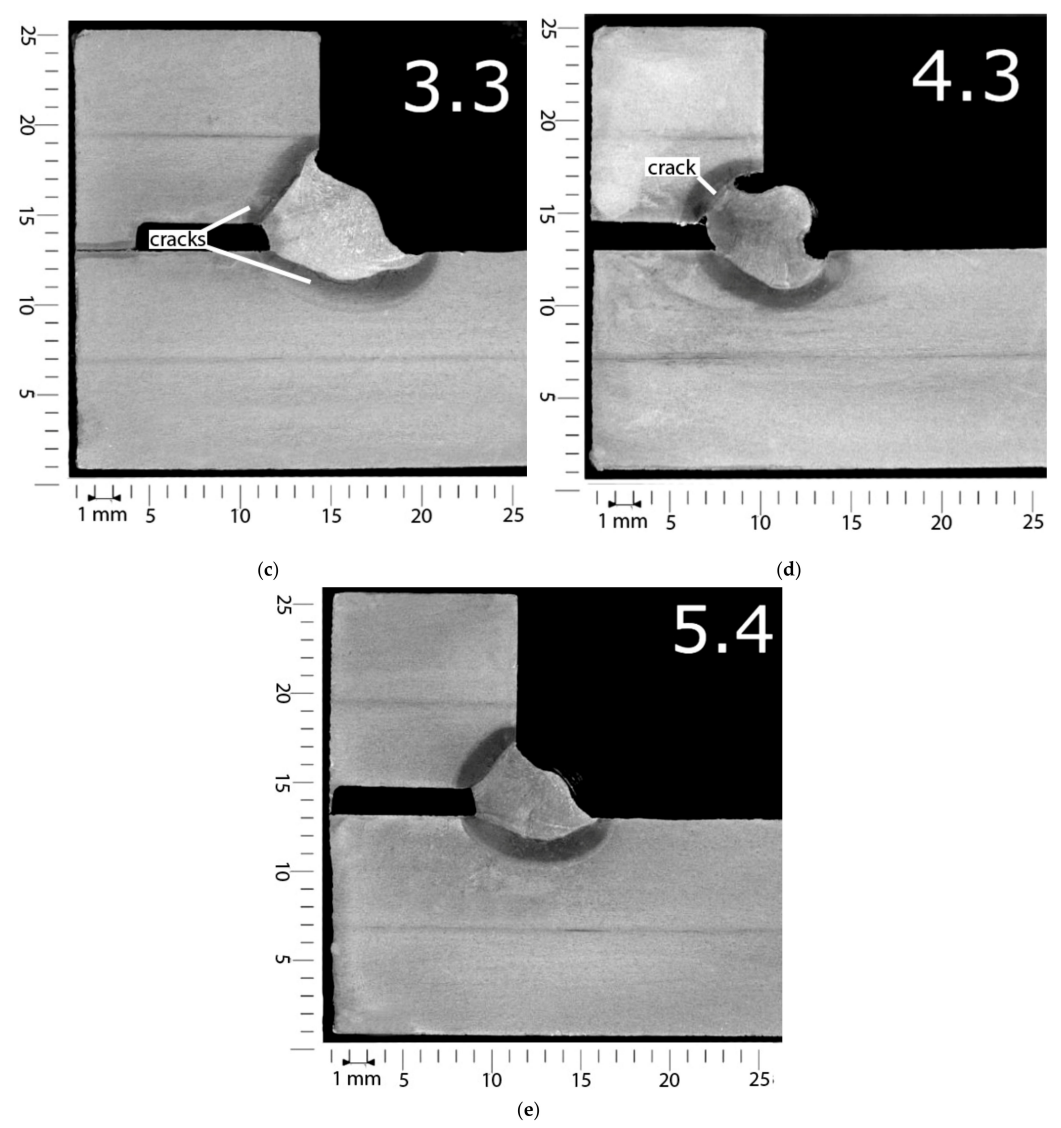
Exemplary results of macroscopic testing: (**a**) weld 1—specimen 1 (non-modified electrode), pores and undercuts; (**b**) weld 1—specimen 2 (underwater electrode), cracks in the heat-affected zone (HAZ); (**c**) weld 2—specimen 3 (concrete impregnate), cracks in the HAZ and undercuts; (**d**) weld 2—specimen 4 (liquid foil), cracks in the HAZ and deep undercuts; (**e**) weld 2—specimen 5 (paraffin wax), no imperfections.

Exemplary macroscopic photos are presented in Figure 3. The macroscopic observations also showed differences in specimens welded with different electrodes. Specimen 1, welded using non-modified electrodes, is characterized by the existence of porosity in the weld, which proved the results of VT (Figure 3a). Additionally, undercuts were observed. However, no cracks occurred. These cracks were observed in weld 1 (Figure 3b), which was performed using underwater electrodes (specimen 2). These cracks were located in the HAZ, parallel to the fusion line. This location is characteristic of cold cracks occurring in wet welding conditions, as stated for the investigated S460N steel in previous investigations [39]. Cracks were observed in joints welded (specimen 3) using electrodes coated with concrete impregnate (Figure 3c). These cracks were longer and wider than in specimen 2. Additionally, small undercuts occurred in two welds of this specimen. The biggest imperfections were observed in specimen 4, performed with the use of liquid-foil-coated electrodes. The quality of both welds was poor, with cracks and deep undercuts observed in each cross-section (Figure 3d). The macroscopic observations of specimen 5 showed no imperfections in three out of four observed cross-sections (Figure 3e). The last cross-section was cut from locations characterized by many imperfections (Figure 2e), so the macroscopic test confirmed the poor quality of the joint in this location.

Macroscopic tests showed the presence of many imperfections in all welded joints, which is typical for underwater conditions [5,7,9]. The most often observed were undercuts and cracks. Garg et al. [50] stated that increasing the cooling rate results in an increasing number of undercuts in electric welded joints. The high cooling rate in wet welding conditions leads to the presence of undercuts in all specimens. The macroscopic tests of CTS specimens showed differences in comparison to the pad weld observations performed in previous investigations [39]. No cracks were observed for pad welds. The presence of cold cracks in joints with filled welds resulted from the different thermal conditions. These differences are connected to the different shapes of the specimens and the presence of notches in CTS samples [42].

### 3.4. Vickers HV10 Hardness Measurements

**Figure 4:**
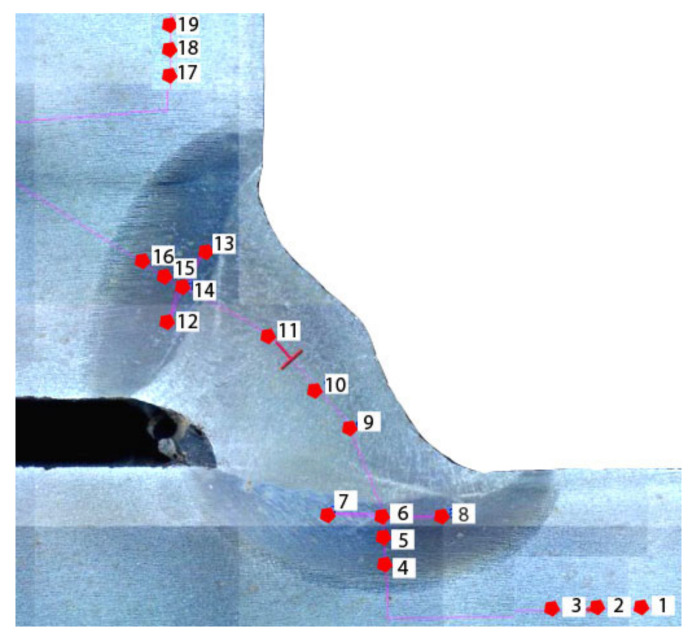
The distribution of measurement points.

Hardness measurements were performed in one cross-section from the test weld at 19 points: 3 in the weld metal, 6 in the BM, and 10 in the HAZ (5 in each side of the joint). A schematic of the distribution of the measurement points is presented in Figure 4.

Following the EN ISO 15614-1:2017 standard [51], S460N steel is classified as a material from group 1.3. In accordance with requirements listed in this document, the maximum HAZ hardness for group 1.3 materials cannot exceed 380 HV10.

**Figure 5:**
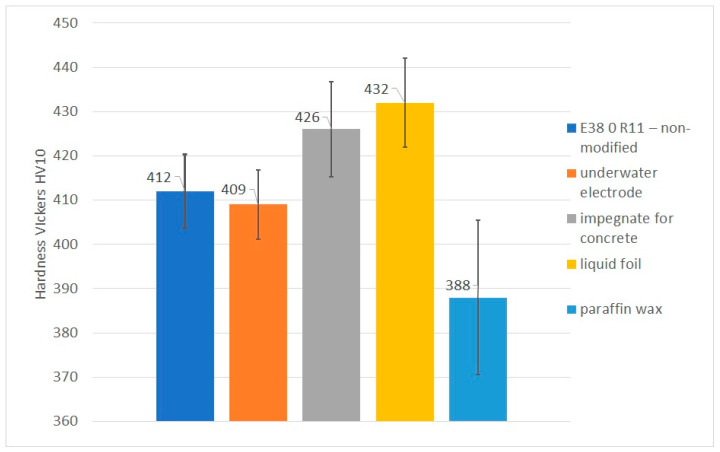
The average HV10 hardness values in the HAZ.

Hardness measurements showed that the HAZ hardness values in specimen 1 (non-modified electrode) and specimen 2 (underwater electrode) were similar, with both being in the range of 400–430 HV10. Previous investigations performed for pad welds [39] showed that the average hardness for non-modified electrodes was 479 HV10 and for underwater electrodes was 472 HV10. This resulted from the greater dilution in pad welds than in welded joints with fillet welds, as stated in the literature by Sun et al. [52]. The same differences between pad welds and CTS specimens were observed for other specimens welded using different electrodes. Additionally, it was observed that usage of concrete impregnate and liquid foil resulted in increased hardness. The hardness of specimen 3 increased to 448 HV 10, while that of specimen 4 increased to 454 HV 10. The measurements of specimen 5, which was welded with electrodes, confirmed the results of previous experiments [39]. The hardness in the HAZ of the joint welded using an electrode with paraffin was significantly lower. Moreover, some measurements were lower than 380 HV10, which is minimum value required for joints welded in air. It had been proven previously [25] that higher-quality S460N steel joints are characterized by lower HAZ hardness. The same effect was observed in the presented investigations. Previous investigations [39] showed that high HAZ hardness could be observed in structures characterized by the presence of a greater number of cold cracks. This suggests that the different hydrophobic coatings investigated in this paper may result in differing susceptibility of HSLA steels to cold cracks. The Vickers HV10 hardness measurement results are listed in Table 5. Values higher than 380 HV10 are shown in bold. It was proven that paraffin wax as a coating on covered electrodes provides significantly decreases the hardness in the HAZ. The average hardness was higher than 380 HV10, however that value is recommended for air welding, in which the cooling rate is much lower than in wet welding. A graphical comparison of average HAZ hardness values is presented in Figure 5.

### 3.5. Metallographic Microscopic Testing

**Figure 6:**
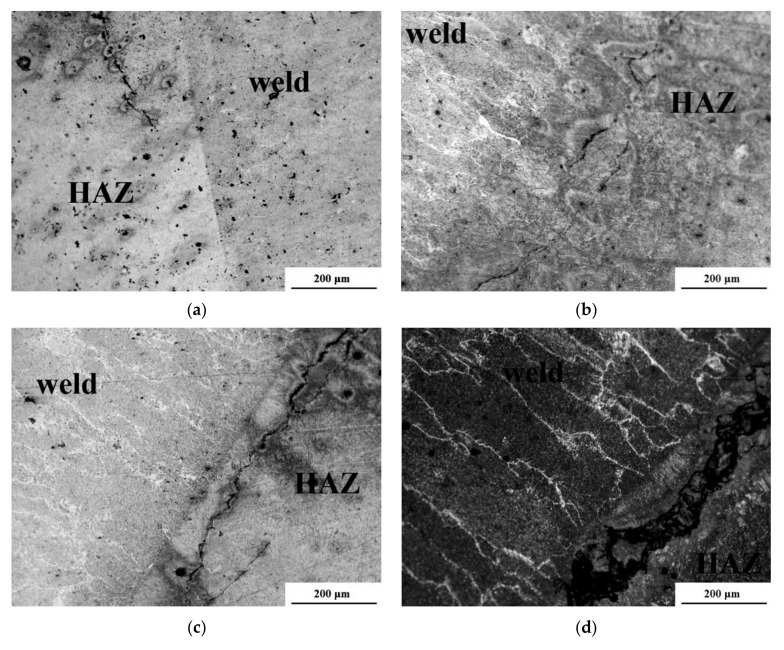

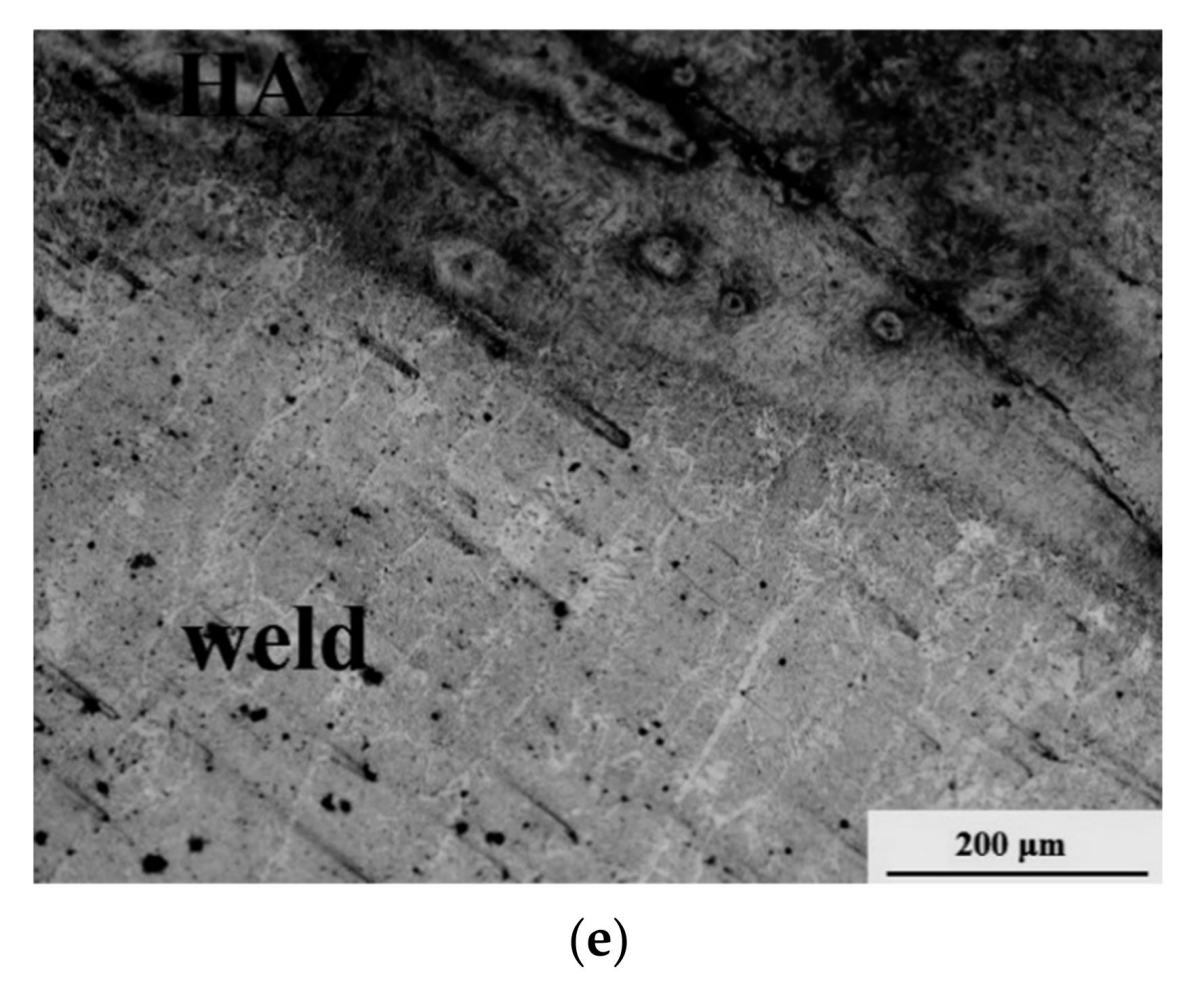
Optical micrographs of: (**a**) specimen 1 (non-modified electrode); (**b**) specimen 2 (underwater electrode); (**c**) scheme 3 (concrete impregnate); (**d**) specimen 4 (liquid foil); (**e**) specimen 5 (paraffin wax).

The microstructures are shown in Figure 6. The main aim of the microscopic test was to evaluate the specimens for the occurrence of cold cracks. It had been proven in previous investigations [39] that the use of electrodes coated with concrete impregnate and liquid foil generated 85–95 mL/100 g diffusible hydrogen in deposited metal. In comparison, the non-modified electrode generated 75 and the underwater electrode 65 mL/100 g diffusible hydrogen. However, the use of paraffin wax decreased the hydrogenation below 50 mL/100 g diffusible hydrogen. These results and the hardness measurements presented in Section 3.3 suggest differences in specimens welded with different filler materials. From each specimen, four cross-sections were observed in the HAZ and in the weld metal. Each joint was characterized by typical microstructures for the underwater-welded HSLA joints [5,9,25]. The weld metal presented a dendritic structure. The HAZ consists of brittle structures such as martensite and bainite. There were no significant microstructural differences in specimens 1–4. In specimen 5, the dimension of coarse-grained martensite area in HAZ was the lowest. Microscopic observations of specimen 1, which was welded with non-modified electrodes, showed long cracks in the HAZ running parallel to the fusion line along 30–40% of its length. (Figure 6a). Additionally, short cracks were found in the weld metal. Lower numbers of cracks were observed in the HAZ of the specimen welded using underwater electrodes (Figure 6b). These cracks ran through 15–20% of the fusion line length. Both specimens were characterized by similar hardness in the HAZ. The differences in the number of cracks resulted from different hydrogen amounts in the deposited metal [38]. No cracks were found in the weld metal. Microscopic tests showed that concrete impregnate as an electrode coating leads to increased numbers of cracks, both in the HAZ and the weld metal (Figure 6c). HAZ cracks ran parallel and vertical to the fusion line. They started in the weld root and propagated along 70–80% of the fusion line length. The worst results were found during observations of test welds in specimen 4 (Figure 6d). Cracks in the HAZ were wide and long (90–100% of the fusion line length). However, the number of cracks in the weld was smaller than in specimen 3. It can be stated that liquid foil and concrete impregnate cannot be used as protective coatings during wet welding of HSLA steels. Microscopic testing of the specimen welded using electrodes coated with paraffin wax showed much different results than for other hydrophobic substances. No cracks were found in the weld metal. In the HAZ, only one short crack running parallel to the fusion line was found (Figure 6e). Microscopic observations proved that cold cracks occurred in wet-welded joints. A number of cold cracks resulted from the presence of brittle structures in the HAZ, characterized by high hardness, with the highest amount of diffusible hydrogen in the deposited metal [39]. It was also proven that HSLA steels are materials in which the high cooling rate leads to creation of the brittle martensitic microstructure [53].

## 4. Conclusions

The effects of different hydrophobic substances used to cover electrodes on the weldability of the HSLA S460N steel were assessed. It was proven that paraffin wax, as a waterproof substance, could improve the weldability of offshore S460N steel welded in water.

The obtained results allowed the following main conclusions to be drawn:

1. The investigated S460N steel was characterized by cold cracking susceptibility in the water. Specimens welded using commercial general-use electrodes and electrodes for underwater applications were characterized by the presence of cold cracks in the HAZ. The HAZ hardness exceeded 420 HV10;
2. The usage of paraffin wax as a protective coating on the surface of the filler material improved the weldability of the investigated material. The HAZ hardness values decreased by 30–40 HV10, and at many measurement points such values were lower than 380 HV10, which is the required level for S460N steel air-welded joints;
3. The concrete impregnate and liquid foil, which are widely used as protective waterproof coatings, cannot be used to improve the weldability of steel. Both substances provide a significant increase of the HAZ hardness of wet-welded specimens (above 440 HV10). Additionally, both substances lead to increased susceptibility of the investigated steel to cold cracking;
4. The paraffin wax leads to a decreased number of imperfections, such as pores and undercuts, which are typical of underwater conditions;
5. The next step in investigations of the usage the waterproof coatings should be testing the mechanical properties of underwater-welded joints with butt welds and fillet welds.

## Figures and Tables

**Table 1:** The chemical compositions of the used materials (wt.%).

Material	C	Mn	Si	Ni	Cr	P	Cu	Ce_IIW_^3^
S460N ^1^	0.16	1.51	0.53	0.05	0.07	0.020	0.13	0.464
E38 0 R11 electrode ^2^	0.07	0.55	0.44	-	0.04	0.010	0.05	-
E42 2 1Ni RR 51 electrode ^2^	0.05	0.50	0.45	0.30	-	0.025	-	-

^1^ In accordance with emission spectrometry with spark excitation analysis. ^2^ Following manufacturers’ data. ^3^ Carbon equivalent as per International Institute of Welding.

**Table 2:** The mechanical properties of the used materials following the manufacturers’ data.

Material	Yield Point, R_e_ (MPa)	Tensile Strength, Rm (MPa)	Elongation, A5 (%)
S460N	511	626	27.3
E38 0 R11 deposit	503	538	26.0
E42 2 1Ni RR 51 deposit	-	540	26.0

**Table 3:** Description of the used hydrophobic substances.

Type of Hydrophobic Substance	Composition
Impregnate for concrete	Silane-siloxane resins based.
Liquid foil	1,2-benzisothiazol-3(2H)-on, 3:1 isothiazoline mixture.
Paraffin wax	Mixture of hydrocarbon molecules containing between twenty and forty carbon atoms.

**Table 4:** The welding parameters.

Specimen No.	Electrode Condition	Weld	I (A)	U (V)	Vsp (mm/s)	ql (kJ/mm)
1	E38 0 R11—non modified	1	161	24.0	4.21	0.92
		2	161	21.0	3.64	0.93
2	Underwater electrode	1	160	27.7	4.23	1.05
		2	160	28.5	3.84	1.19
3	Impregnate for concrete	1	160	26.0	4.55	0.92
		2	160	28.5	3.71	1.21
4	Liquid foil	1	159	27.5	3.59	1.23
		2	159	27.5	3.99	1.10
5	Paraffin wax	1	161	20.7	3.67	0.91
		2	160	22.0	3.33	1.06

**Table 5:** The results of Vickers HV10 measurements.

Sample	BM			HAZ					Weld			HAZ					BM		
	1	2	3	4	5	6	7	8	9	10	11	12	13	14	15	16	17	18	19
1.1.	179	180	179	**400**	**401**	**430**	**422**	**417**	294	296	319	**421**	**410**	**426**	**406**	**408**	184	181	184
1.3.	176	180	178	**405**	**405**	**410**	**411**	**420**	252	259	256	**413**	**418**	**411**	**405**	**406**	183	181	186
2.1.	174	176	174	**401**	**402**	**408**	**409**	**407**	264	271	267	**416**	**413**	**408**	**405**	**408**	177	176	174
2.3.	180	182	180	**403**	**404**	**427**	**430**	**413**	238	246	258	**409**	**409**	**409**	**403**	**401**	180	184	184
3.1.	188	188	187	**408**	**430**	**433**	**431**	**448**	255	254	249	**426**	**435**	**433**	**418**	**419**	187	190	189
3.3.	188	187	188	**413**	**420**	**443**	**431**	**436**	245	233	238	**421**	**424**	**421**	**419**	**408**	187	185	187
4.1.	187	186	187	**412**	**430**	**440**	**430**	**445**	273	306	279	**433**	**421**	**427**	**411**	**411**	190	190	186
4.3.	185	187	190	**433**	**433**	**446**	**451**	**441**	265	265	272	**454**	**424**	**444**	**426**	**436**	185	189	187
5.1.	180	176	179	**382**	**384**	**408**	**411**	**419**	215	203	203	**405**	**404**	**401**	371	380	178	177	176
5.3.	175	175	177	372	369	**383**	**407**	**399**	237	248	235	379	365	**396**	369	362	179	175	173

## Data Availability

Experimental methods and results are available from the authors.
